# Interaction of diet and physical activity on dementia risk: the Rotterdam study

**DOI:** 10.1016/j.tjpad.2026.100594

**Published:** 2026-05-13

**Authors:** Muhammed Lamin Sambou, M. Arfan Ikram, M. Kamran Ikram, Jeremy A. Labrecque, Frank J. Wolters

**Affiliations:** a Dept. of Epidemiology, Erasmus MC – University Medical Center Rotterdam, Rotterdam, the Netherlands; b Dept. of Radiology & Nuclear Medicine, Erasmus MC – University Medical Center Rotterdam, Rotterdam, the Netherlands

**Keywords:** Physical activity, MIND diet, Interaction, Dementia

## Abstract

**Background:**

Diet and physical activity have been reported as independent risk factors for dementia, but few published studies have investigated the interactive effects of the two on dementia risk. Understanding their synergism could help shape more effective prevention strategies. Therefore, we assessed the potential interactions between MIND diet adherence and physical activity on the long-term risk of incident dementia in the population-based Rotterdam Study.

**Methods:**

Between 2009–2013, 5016 participants of the population-based Rotterdam Study were recruited (mean age 69.76 years, 57.9% women). All participants filled in questionnaires regarding their adherence to the MIND diet and time spent on moderate to vigorous physical activity (Metabolic Equivalent of Task [MET] hours per week). Participants were subsequently followed for incident dementia until January 1, 2021. We applied multivariable Cox regression models to assess interaction on both additive and multiplicative scales.

**Results:**

Median values were 7.5 for the MIND diet score and 28.0 for MET hours per week. Of all 5016 participants, 2718 (54.2%) adhered to a high MIND diet score (≥7.5), and 2513 (50.1%) reached at least 28.0 MET hours per week. During a mean follow-up of 6.6 years, 365 participants (7.8%) developed dementia. Both higher physical activity and higher MIND-diet score were independently associated with a lower risk of dementia, but there was no significant interaction on the additive scale (RERI [95%CI]=-0.42 [-1.26 to 0.12]), nor on the multiplicative scale (HR_interaction_=0.71 [0.46–1.09]). Sex-stratified analysis suggested a negative interaction between exposures in women, but not in men.

**Conclusion:**

Better adherence to the MIND-diet and higher levels of physical activity were each associated with a reduced risk of dementia, but overall there was no indication that their joint effects were greater than the product or sum of their individual parts. Potential sex differences warrant further exploration in different populations.

## 1 Introduction

Dementia is a public health concern, with the number of affected individuals projected to increase to 78 million by 2030 and 139 million by 2050 [1]. Roughly half of all dementia cases are attributable to modifiable risk factors [2]. Among acknowledged modifiable lifestyle factors, diet and physical activity are important candidates for preventative intervention, jointly accounting for approximately 12% of dementia cases [3]. Previous studies indicated that adherence to a higher Mediterranean-type diet and moderate to vigorous physical activity, in particular, are associated with a 35 to 44% relative risk reduction of dementia [3,4]. However, few published studies have investigated the interactive effects of diet and physical activity on dementia risk.

Considering the close behavioral and biological link between diet and physical activity, targeting both in a combined intervention may enhance the effectiveness and efficiency of preventive strategies [5]. For instance, exercise facilitates the absorption of essential amino acids and promotes a healthier gut microbiome [6,7]. Alterations in the gut microbiota may influence how diet affects brain function, potentially contributing to neuroinflammation, neuronal damage, and an increased risk of dementia [8,9]. The MIND diet in particular provides antioxidants, polyphenols, and anti-inflammatory nutrients that support brain health and combat oxidative stress. Together with physical activity, they could help reduce inflammation and reactive oxygen species (ROS) [10,11] and exert positive effects on several dementia-related risk factors, including obesity, high blood pressure, elevated LDL cholesterol, diabetes, and depression [[12], [13], [14], [15], [16]].

Moreover, the relationship between physical activity and adherence to the MIND diet may reflect shared health-conscious habits [17]. Earlier research shows that individuals with higher fitness levels are more likely to comply with dietary recommendations [18], and those who are physically active tend to consume greater amounts of fiber and micronutrients, while limiting their intake of total and saturated fats compared to inactive individuals [19]. A study showed that people who boost their physical activity over a decade tend to also improve their diet quality [20]. This indicates that changes in diet and exercise habits often happen together and represent interconnected, multifaceted behaviors that can mutually support one another.

Therefore, we determined the magnitude of potential additive and multiplicative interactions between MIND diet adherence and physical activity on the long-term risk of incident dementia in a population-based study. Interaction on the multiplicative scale helps assess potential biological effect modification, while interaction on the additive scale is more relevant for public health, as it reflects whether the joint effect of two exposures is greater than the sum of their individual effects. By evaluating both scales, we provide a more comprehensive picture of how the MIND diet and physical activity may jointly influence dementia risk.

## 2 Methods

### 2.1 Study population

**Fig. 1:**
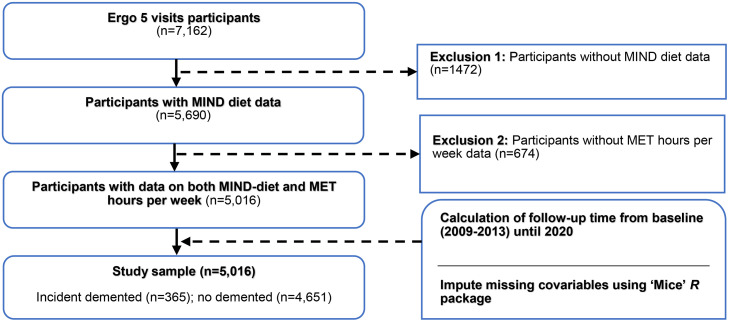
Schematic overview of eligible Rotterdam Study participants.

This research was embedded within the Rotterdam Study, a prospective population-based cohort in the Ommoord district of Rotterdam, the Netherlands. The study currently includes a total of 17,931 participants aged 40 years and older, recruited in four waves since the inception of the cohort in 1990 [21]. Details of the study have been described previously. In brief, participants are assessed every four years, including an extensive home interview and visit to a dedicated research center. For the present analysis, we included all participants who took part in the fifth examination round (2009–2013), which included simultaneous assessment of physical activity and diet. Of 7162 eligible participants, 5016 individuals completed both the food frequency questionnaire and physical activity questionnaire and were included in the analysis (Fig. 1).

### 2.2 Ethics statement

The Rotterdam study has been approved by the Medical Ethics Committee of Erasmus University Medical Center (registration number MEC 02.1015) according to the Population Screening Act, executed by the Dutch Ministry of Health, Welfare, and Sport (license number 1071272-159531-PG). All participants provided written informed consent.

### 2.3 Data availability statement

Requests for use of the data underlying this report can be directed to the Rotterdam Study management team (secretariat.*epi*@erasmusmc.nl) and will be evaluated according to the established data access protocol. Due to privacy regulations and informed consent restrictions, the data cannot be publicly shared in an open repository.

### 2.4 Assessment of physical activity

Physical activity was assessed using the LASA Physical Activity Questionnaire (LAPAQ), which has been reported to have higher quality and greater suitability for the Dutch population than the Zutphen and Becker questionnaires [22]. The LAPAQ contains questions regarding the frequency and duration of walking, cycling, sports, gardening, and housework. Participants were asked how many hours per week they spent in each activity in the past two weeks.

To quantify activity intensity, we used the metabolic equivalent of task (MET). A panel of three assigned MET-values to all activities mentioned in the questionnaire, according to the 2011 updated version of the Compendium of Physical Activities [23]. Finally, we multiplied MET-values of specific activities with time (in hours) per week spent in that activity to calculate MET∙hours∙week in total physical activity. Similar to earlier research, we found that participants in the Rotterdam Study exhibited high physical activity levels [24]. Therefore, we calculated weekly MET hours for moderate to vigorous physical activity in accordance with the WHO’s physical activity guidelines for adults [12].

### 2.5 Assessment of diet

Dietary intake was assessed using a self-administered food frequency questionnaire (FFQ) that included 389 food items, containing questions on frequency and portion sizes of food item consumption over the last month [25]. The FFQ has been validated against other dietary assessment methods, which showed that, based on these FFQs, participants can be adequately ranked according to their food and nutrient intake. Using the FFQ, we calculated the MIND diet score (Mediterranean-DASH Intervention for Neurodegenerative Delay), which has been associated with slower cognitive decline and a lower risk of developing dementia [26].

The MIND diet comprises 15 dietary components, of which 10 are considered beneficial for brain health (green leafy vegetables, other vegetables, nuts, berries, legumes, whole grains, fish, poultry, olive oil, and wine) and 5 are regarded as detrimental (including red meat, butter and stick margarine, cheese, fried or fast food, and pastries or sweets). Olive oil intake was scored as 1 point if it was used as the primary cooking fat more than half the time, and 0 otherwise. For all other components, adherence was scored as 0 points for no adherence, 0.5 points for moderate adherence, and 1 point for full adherence. The scores for each component were then summed to yield the total MIND diet score, ranging from 0 to 15, with higher scores indicating greater adherence to the diet [27].

### 2.6 Ascertainment of dementia

Participants were screened for dementia at baseline and at each follow-up visit using the Mini-Mental State Examination (MMSE) and the Geriatric Mental Status Schedule (GMS) organic level. Individuals scoring below 26 on the MMSE or scoring 0 on the GMS organic level underwent further evaluation using the Cambridge Examination for Mental Disorders in the Elderly diagnostic interview. In addition to scheduled examinations, participants were continuously monitored for dementia through electronic linkage of the study database with medical records from general practitioners and the regional institute for outpatient mental health care. Final diagnoses of dementia were determined by a consensus panel, led by a neurologist, following established criteria for all-cause dementia (DSM-III-R). Dementia follow-up was done until January 1, 2021. Additional details on the dementia assessment procedures have been published previously [21].

### 2.7 Covariable assessment

During the interview, information on the following covariables was collected: age, sex, education, employment status, smoking status, alcohol consumption, depressive symptoms, poor sleep quality, the use of antidiabetic medication, lipid-lowering therapy, and blood pressure-lowering medication. Systolic blood pressure was measured with a random-zero sphygmomanometer on the right arm. Body Mass Index (BMI) was calculated from measured weight and height (kg/m^2^). Serum glucose levels and high-density lipoprotein (HDL) cholesterol were acquired by an automated enzymatic procedure. APOE genotype was assessed using polymerase chain reaction, and stratified into carriers and noncarriers of the APOE-ε4 allele (ε2/ ε2, ε2/ ε3, ε3/ ε3, ε3/ ε4, ε2/ ε4, and ε4/ ε4). For this study, we binarized the variable as APOE ε4- (ε2/ ε2, ε2/ ε3, ε3/ ε3) and APOE ε4 + (ε3/ ε4, ε2/ ε4, ε4/ ε4).

### 2.8 Statistical analysis

Descriptive statistics were performed to summarize baseline characteristics of the study participants. Multivariate imputation of missing covariates was done using the ‘mice’ R package (with 50 iterations). Covariate balance was evaluated through the calculation of propensity scores. Participants were censored if they had dementia, died, or were lost to follow-up at the end of follow-up.

We then applied multivariable-adjusted Cox proportional hazards models to estimate the independent associations of the MIND diet and physical activity with incident dementia, with each exposure adjusted for the other. The separate effects of the MIND diet score and moderate-vigorous MET hours per week were examined both on continuous and categorized scales.

We also analyzed both exposures in their standardized forms, all conditional on the covariables. The proportional hazards assumption of the Cox model with interaction was checked through visual inspection of Schoenfeld residuals (supplementary Fig. S1).

We assessed additive interaction between the two continuous exposures on incident dementia by calculating the Relative Excess Risk due to Interaction (RERI), Attributable Proportion (AP), and Synergy Index (SI), along with 95% confidence intervals obtained via bootstrapping. A significant RERI or AP above 0 was interpreted as evidence of positive additive interaction, while values below 0 indicated a negative interaction. Similarly, an SI greater than 1 was taken to suggest the presence of an interaction effect. The different measures of additive interaction (RERI, AP, S) help triangulate and confirm the presence and strength of interaction on the additive scale, providing a more nuanced understanding [28]. To further explore the additive interaction effects, we categorized the MIND diet and physical activity variables using median thresholds. Confidence intervals for these estimates were derived using the Method of Variance Estimates Recovery (MOVER), as it outperforms the delta method at typical sample sizes and shows comparable performance to bootstrapping with larger samples [29].

To assess multiplicative interaction between the exposures on a continuous scale, we used a Cox proportional hazards model that included an interaction term. We centered the exposure variables by subtracting their respective means from each value. This approach helps minimize the correlation between the interaction term and the main effects, resulting in more stable coefficient estimates [30]. A hazard ratio (HR) of the interaction term significantly greater than 1 indicates a positive (synergistic) interaction, while an HR less than 1 suggests a negative (antagonistic) interaction. Multiplicative interaction assesses whether the combined effect of two exposures on a relative scale (e.g., hazard ratio) is different from what is expected, while additive interaction focuses on the absolute risk difference, which is often more relevant for public health impact. As a sensitivity analysis, we repeated the interaction analysis without some covariates that could theoretically be argued as mediators (glucose level, BMI, systolic blood pressure, non-HDL cholesterol, and sleep quality).

Finally, we performed stratified analysis to explore whether the interaction effects differed by sex (women vs. men) and by level of education attained. For this analysis, we coded ‘primary’ and ‘lower/intermediate’ education as ‘lower education,’ while ‘intermediate vocational’ and ‘higher education’ categories were considered ‘higher education.’ We calculated the E-value for the main finding and the sex-stratified results to estimate the minimal level of association that an unmeasured confounder must have with the exposure and outcome to entirely explain away the observed effect. The reporting of the interaction results was in accordance with the STROBE guidelines (Supplementary Table S7) [28].

All analyses were conducted in R version [4.4.2], using the ‘ppcor,’ ‘Table 1,’ ‘cobalt,’ ‘WeightIt,’ ‘CarefullyCausal,’ ‘ggplot2,’ ‘epiR,’ ‘survival,’ ‘survminer,’ ‘dplyr,’ ‘gmodels,’ ‘mice,’ ‘interactionR,’ and ‘EValue’ packages. Alpha was set at 0.05.

**Table 1:** Baseline characteristics of the study population (n = 5016).

Variables	Total, N (%)	MIND Diet		Physical activity	
		MIND Diet <7.5	MIND Diet ≥7.5	MET h/week < 28.0	MET h/week ≥ 28.0
Number of participants	5016 (100%)	2298 (45.8%)	2718 (54.2%)	2503 (49.9%)	2513 (50.1%)
Age, Mean± SD	69.76±8.73	71.0 ± 8.9	68.7 ± 8.5	71.3 ± 9.3	68.2 ± 7.9
Sex					
Male	2114 (42.1%)	1088 (47.4%)	1026 (37.8%)	1020 (40.8%)	1094 (43.5%)
Female	2902 (57.9%)	1210 (52.7%)	1692 (62.3%)	1483 (59.2%)	1419 (56.5%)
**Education**					
Primary	392 (7.8%)	216 (9.4%)	176 (6.5%)	221 (8.8%)	171 (6.8%)
Lower/intermediate	2001 (39.9%)	987 (43.0%)	1014 (37.3%)	1051 (42.0%)	950 (37.8%)
Intermediate vocational	1518 (30.3%)	717 (31.2%)	801 (29.5%)	719 (28.7%)	799 (31.8%)
Higher	1105 (22.0%)	378 (16.5%)	727 (26.8%)	512 (20.5%)	593 (23.6%)
**Smoking status**					
Non-smoker	4465 (89.0%)	1980 (86.2%)	2485 (91.4%)	2198 (87.8%)	2267 (90.2%)
Current smokers	549 (10.9%)	317 (13.8%)	232 (8.5%)	304 (12.2%)	245 (9.8%)
Total alcohol, Mean± SD	7.40±8.09	7.55±8.60	7.28±7.63	6.96±8.05	7.85±8.11
**Employment status**					
Retired or unemployed	4125 (82.2%)	1900 (82.7%)	2225 (81.9%)	2050 (81.9%)	2075 (82.6%)
Employed	886 (17.7%)	398 (17.3%)	488 (18.0%)	451 (18.0%)	435 (17.3%)
**Sleep quality (PSQI)**					
Good sleep (PSQI<5)	3648 (72.7%)	1633 (71.1%)	2015 (74.1%)	1760 (70.3%)	1888 (75.1%)
Poor sleep (PSQI≥5)	1368 (27.3%)	665 (28.9%)	703 (25.9%)	743 (29.7%)	625 (24.9%)
**Depressive symptoms**					
No depression (CESD<16)	4603 (91.8%)	2076 (90.3%)	2527 (93.0%)	2250 (89.9%)	2353 (93.6%)
Depression (CESD≥16)	413 (8.2%)	222 (9.7%)	191 (7.0%)	253 (10.1%)	160 (6.4%)
**APOE ε4 carrier**					
APO ε4-	3641 (72.6%)	1691 (73.6%)	1950 (71.7%)	1834 (73.3%)	1807 (71.9%)
APO ε4+	1375 (27.4%)	607 (26.4%)	768 (28.3%)	669 (26.7%)	706 (28.1%)
Body Mass Index (kg/m^2^)	27.46±4.31	27.6 ± 4.3	27.4 ± 4.3	28.1 ± 4.6	26.9 ± 3.9
Glucose levels, Mean± SD	5.79±1.23	5.86±1.32	5.73±1.14	5.87±1.30	5.71±1.16
Non-HDL cholesterol	1.49±0.42	1.45±0.42	1.53±0.43	1.44±0.41	1.54±0.44
**Antidiabetic medication**					
No	4569 (91.1%)	2066 (89.9%)	2503 (92.1%)	2223 (88.8%)	2346 (93.4%)
Yes	447 (8.9%)	232 (10.1%)	215 (7.9%)	280 (11.2%)	167 (6.7%)
**Lipid-lowering therapy**					
No	3474 (69.3%)	1541 (67.1%)	1933 (71.1%)	1678 (67.0%)	1796 (71.5%)
Yes	1542 (30.7%)	757 (32.9%)	785 (28.9%)	825 (33.0%)	717 (28.5%)
Systolic blood pressure, Mean± SD	144.63±22.20	146±22.0	144±22.3	146±22.7	143±21.6
**Blood pressure-lowering medication**					
No	4982 (99.3%)	2275 (99.0%)	2707 (99.6%)	2485 (99.3%)	2497 (99.4%)
Yes	34 (0.7%)	23 (1.0%)	11 (0.4%)	18 (0.7%)	16 (0.6%)

Notes: SD=standard deviation, N=number of participants; PSQI, Pittsburgh Sleep Quality Index; CESD, Center for Epidemiologic Studies Depression Scale; HDL=high density lipoprotein.

Percentage missingness in the covariates: APOE, 6.24%; alcohol, 0.36%; smoking, 0.14%; systolic blood pressure, 1.48%; glucose in serum, 2.61%; Non HDL cholesterol, 2.61%; BMI, 0.18%; poor sleep quality, 4.19%; depression, 0.34%; education, 1.16%; employment status, 33.29%; antidiabetic medication, 0.12%; lipid lowering therapy, 0.12%; blood pressure lowering medication, 0.12%; anti-inflammatory medication, 0.12%.

## 3 Results

Baseline characteristics of the study population are presented in Table 1. Of all 5016 participants (mean age: 69.8 ± 8.7 years), 2902 (57.9%) were women. Mean BMI was 27.5 ± 4.3 kg/m^2^. The median physical activity level was 28.0 MET hours per week, and the median MIND diet score was 7.5 (scale: 0–15). Overall, 2718 participants (54.2%) adhered to a high MIND diet score (≥7.5), and 2513 (50.1%) achieved at least 28.0 MET-hours per week. The estimated effect of the covariates on dementia risk is reported in Supplementary Table S5.

For physical activity, the risk of dementia was lower in the second tertile (HR = 0.83; 95% CI: 0.66–1.06) and in the third tertile (HR = 0.71; 95% CI: 0.53–0.95) compared with the first tertile (Table 2). Similar results were observed for MIND diet score (second tertile: HR [95% CI] = 0.72 [0.57–0.92]; third tertile: 0.58 [0.45–0.76]). A 1-unit increase in the MIND diet score was associated with a 14% decreased hazard of incident dementia in the fully adjusted model (HR= 0.86; 95% CI: 0.81–0.93). Comparing the standardized effect sizes for both exposures, estimates were somewhat stronger for the MIND diet score than for physical activity (HR [95% CI] per 1-SD increase, for MIND: = 0.79 [0.71- 0.88]; and for physical activity: 0.90 [0.79, 1.03]).

**Table 2:** Physical activity, MIND diet, and incident dementia.

Predictors	N _cases_/N _total_	Model 1		Model 2	
		HR (95%CI)	*P*-value	HR (95%CI)	*P*-value
**Physical activity (MET hours/week)**					
Continuous, per 1 MET-hour/week increase	365/5016	0.99 (0.99- 1.00)	0.39	0.99 (0.99- 1.00)	0.14
Continuous, per 1-SD increase		0.94 (0.83- 1.08)	0.39	0.90 (0.79- 1.03)	0.14
**Tertile**					
T1-Low (<17.5)	174/ 1672	1.0		1.0	
T2- Moderate (1.5- 43.6)	117/ 1672	0.89 (0.70- 1.13)	0.34	0.83 (0.66- 1.06)	0.14
T3-High (≥43.7)	74/ 1672	0.82 (0.61- 1.09)	0.17	0.71 (0.53- 0.95)	**0.02**
**MIND-Diet score**					
Continuous, per 1 point increase	365/5016	0.87 (0.81- 0.93)	**<0.001**	0.86 (0.81- 0.93)	**<0.001**
Continuous, per 1-SD increase		0.80 (0.71- 0.89)	**<0.001**	0.79 (0.71- 0.88)	**<0.001**
Tertiles					
T1-Low (<6.5)	173/ 1672	1.0		1.0	
T2- Moderate (6.5- 8.0)	108/ 1672	0.72 (0.56- 0.91)	**0.01**	0.72 (0.57- 0.92)	**0.01**
T3-High (≥8.1)	84/ 1672	0.58 (0.45- 0.76)	**<0.001**	0.58 (0.45- 0.76)	**<0.001**

Interaction model (centered).

Measure of interaction on the additive scale:.

RERI (95% CI): -0.42 (-1.26, 0.17);.

AP (95% CI): -0.26 (-0.75, 0.13);.

Synergy index (95% CI): 0.59 (0.22, 1.59).

Measure of interaction on multiplicative scale (model-centered): HR (95% CI)= 0.97 (0.80, 1.19).

Notes: HR=Hazard ratio; CI=Confidence interval; MET=Metabolic Equivalent of Task; SD=Standard deviation.

Model 1 includes age, sex, education, and MIND diet/physical activity.

Model 2 includes age, sex, education, employment status, glucose levels, BMI, smoking status, alcohol consumption, MIND diet/physical activity, systolic blood pressure, non-High density lipoprotein cholesterol, APOE4, depressive symptoms, poor sleep quality, antidiabetic medication, lipid-lowering therapy, and blood pressure-lowering medication.

Assessing diet and physical activity as continuous measures, we did not find statistically significant interaction effects on incident dementia, neither on the additive scales (RERI = -0.42; 95% CI: -1.26, 0.17; AP = -0.26; 95% CI: -0.75, 0.13; SI = 0.59; 95% CI: 0.22, 1.59) nor on the multiplicative scale (HR = 0.97; 95% CI: 0.80, 1.19) (Table 2). Similar findings were observed when the exposures were dichotomized based on median cutoffs (Table 3). Moreover, the results from the models excluding variables considered potential mediators were consistent with the main findings (Table S1). The E-value for the point estimate of the combined effect of MIND diet score and physical activity on dementia risk was 1.21 (Supplementary Fig. S2), suggesting a relatively small degree of unmeasured confounding would be sufficient to explain away the observed effect estimates.

**Table 3:** Interaction between MIND diet score and MET hours per week on dementia risk.

MIND Diet	Physical activity (MET hours/week)				Within strata of MIND Diet
	MET hrs./wk. ≥28		MET hrs./wk. < 28		
	N _cases_/N _total_	HR (95%CI)	N _cases_/N _total_	HR (95%CI)	
MIND Diet ≥7.5	58/1474	1.0	95/1244	1.34 (0.96, 1.88)	1.34 (0.96, 1.88)
MIND Diet <7.5	76/1039	1.69 (1.19, 2.39)	136/1259	1.61 (1.16, 2.22)	0.95 (0.71, 1.28)
Within strata of MET hrs./wk.		1.69 (1.19, 2.39)		1.20 (0.92, 1.56)	

Measure of interaction on the additive scale:.

RERI (95% CI): -0.42 (-1.26, 0.12);.

AP (95% CI): -0.26 (-0.73, 0.09);.

Synergy index (95% CI): 0.59 (0.31, 1.14).

Measure of interaction on multiplicative scale: HR (95% CI)= 0.71 (0.46, 1.09).

Results are presented for the fully adjusted model 2, which includes age, sex, education, employment status, glucose levels, BMI, smoking status, alcohol consumption, systolic BP, non-HDL cholesterol, APOE4, depressive symptoms, poor sleep quality, antidiabetic medication, lipid-lowering therapy, and antihypertensive.

In sex-stratified analyses, hazard ratios were higher among women than men. Compared to participants with both higher MIND diet score (≥7.5) and higher physical activity level (≥28.0 MET hours/week), women with low levels of both exposures demonstrated a substantially elevated hazard of dementia (HR=1.82; 95%CI: 1.18, 2.80). The corresponding estimate among men was more modest and did not reach statistical significance (HR=1.38; 95%CI: 0.84, 2.26; ratio of hazard ratios: 1.32). Furthermore, we observed a significant negative interaction between MIND diet score and physical activity in women but not in men (Table 4). The E-value for the sex-stratified interaction results among women indicates that an unmeasured confounder would need a 3.67-fold association with both the exposures and outcome to explain away this result (Supplementary Fig. S2). A significant negative interaction was observed among participants with lower education, but not among those with higher education (Supplementary Table S4).

**Table 4:** Sex-stratified interaction between MIND Diet and MET hours per week on dementia risk.

FEMALE (N = 2902)						MALE (N = 2114)				
MIND Diet	Physical activity (MET hours/week)				Within strata of MIND Diet	Physical activity (MET hours/week)				Within strata of MIND Diet
	MET hrs./wk. ≥28		MET hrs./wk. < 28			MET hrs./wk. ≥28		MET hrs./wk. < 28		
	N_cases_/N _total_	HR (95%CI)	N _cases_/N _total_	HR (95%CI)		N _cases_/N _total_	HR (95%CI)	N _cases_/N _total_	HR (95%CI)	
MIND Diet ≥7.5	32/ 913	1.0	64/ 779	1.59 (1.03, 2.46)	1.59 (1.03, 2.46)	26/ 561	1.0	31/ 465	1.06 (0.62, 1.81)	1.06 (0.62, 1.81)
MIND Diet <7.5	46/ 506	2.43 (1.54, 3.84)	84/ 704	1.82 (1.18, 2.80)	0.75 (0.51, 1.10)	30/ 533	1.01 (0.59, 1.73)	52/ 555	1.38 (0.84, 2.26)	1.36 (0.86, 2.15)
Within strata of MET hrs./wk.		2.43 (1.54, 3.84)	1.14 (0.82, 1.60)				1.01 (0.59, 1.73)		1.30 (0.83, 2.03)	
Measure of interaction on the additive scale: RERI (95% CI): -1.20 (-2.86, -0.30); AP (95% CI): -0.66 (-1.42, -0.14); Synergy index (95% CI): 0.41 (0.21, 0.79). Measure of interaction on multiplicative scale: HR (95% CI)= 0.47 (0.27, 0.83).						Measure of interaction on additive scale: RERI (95% CI): 0.30 (-0.69, 0.95); AP (95% CI): 0.22 (-0.43, 0.71); Synergy index (95% CI): 5.0 (0, inf.). Measure of interaction on multiplicative scale: HR (95% CI)= 1.28 (0.64, 2.56).				

Adjusted model 2: age, education, employment status, glucose levels, BMI, smoking status, alcohol consumption, systolic BP, non-HDL cholesterol, APOE4, depressive symptoms, poor sleep quality, antidiabetic medication, lipid lowering therapy, and antihypertensive.

## 4 Discussion

Adherence to a high MIND diet and higher levels of moderate to vigorous physical activity were independently associated with a reduced risk of incident dementia, but there was no indication of statistical interaction between the two exposures. Sex-stratified results suggested differential interaction between both exposures in women compared to men.

Our study suggests that the combined effects of MIND diet adherence and physical activity in relation to dementia risk do not surpass the sum of their separate effects. This aligns with the findings of Scarmeas et al. (2009), who reported that the protective benefits of the Mediterranean diet and moderate to vigorous physical activity on Alzheimer’s disease risk function independently [3]. In contrast, previous research has highlighted potential complementary behaviors and shared biological mechanisms linking diet and exercise [6]. For instance, Timmerman et al. (2012) showed that a single bout of aerobic exercise can improve blood flow and muscle perfusion, enhancing the uptake and use of essential amino acids during nutrient intake in older adults [7]. Similarly, individuals with higher fitness levels were more likely to comply with dietary recommendations [18], and those who are physically active tend to consume greater amounts of fiber and micronutrients, while limiting their intake of total and saturated fats compared to inactive individuals [19]. This discrepancy suggests that while diet and exercise each play important roles in dementia prevention, their effects may operate independently rather than synergistically. As such, it is unlikely that the effect of combining both in a multidomain trial outweighs the effect of what would be observed in trials addressing each component individually.

In sex-stratified analysis, physical activity and diet appeared to work in a negative interactive way in women only, meaning that the benefit gained from combining both a high-quality MIND diet and regular physical activity was smaller than what would be expected if their individual effects were added together or multiplied. The causes underlying this somewhat unexpected finding remain speculative. Women engaging in high physical activity but poor dietary habits may represent a distinct risk profile, where other unmeasured lifestyle or metabolic factors (such as chronic inflammation, cardiometabolic burden, or hormonal factors) may attenuate the expected protective effect of physical activity. No such interaction was detected in men. However, until further replication, we believe these observations should be treated solely as hypothesis-generating. As such, this finding should not discourage combined healthy behavior, as other health outcomes (e.g., cardiovascular diseases, diabetes, microbiota) may still benefit from joined lifestyle interventions [6,31]. Rather, these results highlight the importance of promoting at least one of these protective behaviors in women who may face barriers to adopting both.

Prior research has also suggested that sex may act as an effect modifier in the diet–physical activity–dementia relationship [32]. Several mechanisms may underlie this differential impact. First, hormonal differences may interact with lifestyle factors in ways that amplify the benefits of diet and exercise in women. For example, estrogen has been shown to influence brain plasticity, cerebral blood flow, and inflammatory processes, which are also modifiable through diet and physical activity [33]. The synergy among these pathways may enhance cognitive resilience in women who adhere to healthier lifestyles. Moreover, a study among young adults from 23 countries reported that women were often more likely than men to adopt and sustain health-promoting behaviors such as balanced dietary patterns and regular physical activity [34]. Another study reported that older women participating in aerobic exercise programs tend to experience more significant cognitive improvements than their male counterparts [35,36]. Findings from the Health, Aging, and Body Composition study also indicated that sustained physical activity over a decade was linked to less cognitive decline across multiple domains in women compared to men [37]. Thus, women may experience a stronger cumulative benefit from concurrent adherence to both behaviors. Additionally, differences in body composition, metabolic regulation, and stress responsiveness may further modify how diet and exercise influence dementia risk across sexes [38]. These observations suggest that sex-specific strategies could be valuable in designing dementia prevention programs.

It is worth nothing that the Rotterdam Study participants reported relatively high physically activitiy, with the majority (95.6%) exceeding the WHO-recommended threshold for moderate-to-vigorous activity (3 MET hours/week). Given this distribution, the median value (28.0 MET-hours/ week) was selected as the cut-off for analysis, in line with earlier cross-cohort harmonization studies on physical activity. While the physical activity questionnaire encompasses a wide range of daily life activities and this carries some potential for overreporting, the within-cohort associations observed in the Rotterdam Study remain consistent with those reported across studies, lending confidence to the validity of our findings [21,39]. While we used regression-based methods to answer a causal question through traditional epidemiological methodology, it is worth acknowledging the growing body of computational and data-driven research that complements such approaches. Recent advances in machine learning and deep learning may offer new possibilities for detecting intereaction patterns in large datasets. Future studies combining lifestyle-based epidemiological findings, such as those presented here, with these advanced computational methods may further strengthen our understanding of modifiable risk factors in dementia research.

Strengths of the current study include the large cohort size with meticulous long-term follow-up for dementia. Several limitations must also be acknowledged. First, both diet and physical activity were assessed using self-reported questionnaires, which may be susceptible to misclassification. Participants may have inaccurately recalled or misreported their dietary intake and physical activity levels, whether due to memory limitations, social desirability bias, or a tendency to overestimate healthy behaviors and underestimate unhealthy ones. Moreover, one-time measurements of both physical activity and diet, which may not fully capture changes in behavior over time or account for prior exposures, potentially introduce exposure misclassification and attenuate the observed associations towards the null. Repeated measures of physical activity and dietary intake across follow-up would provide a more accurate representation of long-term lifestyle patterns and their relationship with dementia. Furthermore, the physical activity questionnaire focuses on specific types of activities and may not account for context (e.g., occupational vs. recreational exercise). These limitations raise concerns about the ‘consistency assumption’ in causal inference, which requires that the ‘treatment’ being studied (e.g., adherence to the MIND diet or a certain level of physical activity) is well-defined and uniform across individuals. In reality, there may be multiple ways participants achieve the same score, some of which may differ meaningfully in their biological or behavioral effects [40]. Future research using more objective and comprehensive measures, such as biomarkers or accelerometer-based physical activity data, could help strengthen the validity of observations. Second, reverse causality cannot be ruled out because individuals in the preclinical stages of dementia may already exhibit reduced physical activity and changes in dietary habits [41]. Although we adjusted for a range of covariables, residual confounding from cognitive engagement, chronic stress, and social activity and support remains a possibility. The E-value indeed underlines the finding is not necessarily robust to unmeasured confounding. Finally, the associations observed in this cohort may not directly translate to other populations where dietary patterns, physical activity behaviors, or measurement tools differ. Also, the study population was predominantly of European ancestry, potentially hampering generalizability to other populations.

In conclusion, better adherence to the MIND-diet and higher levels of physical activity were each associated with a reduced risk of dementia, with no indication that their joint effects were greater than the product or sum of their estimated individual effects. Whether prevention strategies on diet and physical activity should be tailored differently to women and men warrants further study.

## Disclosures

None.

## Declaration of the use of generative AI and AI-assisted technologies in scientific writing and in figures, images, and artwork

We used Claude, version 2025, only for a more concise rewriting of a couple of sentences in the discussion section of the manuscript, which did not affect the formulation of the conclusions and did not require the sharing of any research data with the algorithm.

## Funding

This work is part of the BIRD-NL consortium funded by the Dutch Medical Research Council (ZonMw) as part of the National Dementia Strategy 2021- 2030 by the Dutch Ministry of Health, Welfare and Sport (grant number: 1051003210005)

## CRediT authorship contribution statement

**Muhammed Lamin Sambou:** Writing – review & editing, Writing – original draft, Methodology, Formal analysis, Data curation, Conceptualization. **M. Arfan Ikram:** Writing – review & editing, Supervision, Methodology, Funding acquisition, Data curation, Conceptualization. **M. Kamran Ikram:** Writing – review & editing, Methodology, Data curation. **Jeremy A. Labrecque:** Writing – review & editing, Supervision, Methodology, Conceptualization. **Frank J. Wolters:** Writing – review & editing, Supervision, Funding acquisition, Data curation, Conceptualization.

## Declaration of your competing interest

The authors have no competing interests to declare. Our affiliation or funder does not influence the content of the manuscript or the choice of journal for this study.

## Appendix Supplementary materials

Image, application 1
