# Supplementary material for: Interaction of diet and physical activity on dementia risk: the Rotterdam study
Source: J Prev Alzheimers Dis. 2026 May 13;13(7):100594. doi: 10.1016/j.tjpad.2026.100594 (PMC13196504; doi:10.1016/j.tjpad.2026.100594)
Supplement: Supplementary file 1 [file mmc1.docx]

**Supplementary material**

**Interaction of Diet and Physical Activity on Dementia Risk: the Rotterdam Study**

Muhammed Lamin Sambou^1^, M. Arfan Ikram^1^, M. Kamran Ikram^1^, Jeremy A. Labrecque*^1^, Frank J. Wolters*^1,7^

^1^Dept. of Epidemiology, Erasmus MC – University Medical Center Rotterdam, Rotterdam, the Netherlands

^7^Dept. of Radiology & Nuclear Medicine, Erasmus MC – University Medical Center Rotterdam, Rotterdam, the Netherlands

* senior authorship is shared by drs. Wolters and Labreque

Corresponding author: Frank J. Wolters

Dr. Molewaterplein 40, PO Box 2040, 3000 CA Rotterdam

Email: [f.j.wolters@erasmusmc.nl](mailto:f.j.wolters@erasmusmc.nl)

Phone: +31 107 034 625

**Outline**

Figure S1. Plot showing proportional hazards assumption was satisfied for both exposure variables centered around their mean values (*P*=0.50).

Figure S2. E-value for main results and sex-stratifed analysis.

Table S1. Sensitive analysis of the interaction between MIND diet and MET hours per week on dementia risk.

Table S2. Baseline characteristics of the study population stratified by Sex

Table S3. Sensitive analysis among participants with follow-up time of ≥ 3 years (N=4621)

Table S4. Stratified interaction between MIND Diet and MET hours per week on dementia risk based on education level

Table S5. Estimated effect of covariates on incident dementia

Table S6. STROBE Statement—checklist of items that should be included in reports of observational studies


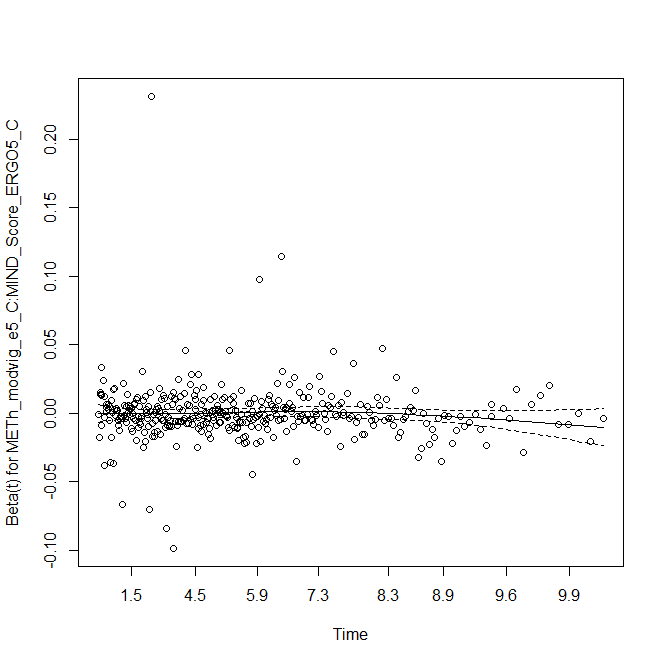


Figure S1. Plot showing proportional hazards assumption was satisfied for both exposure variables centered around their mean values (*P*=0.50).


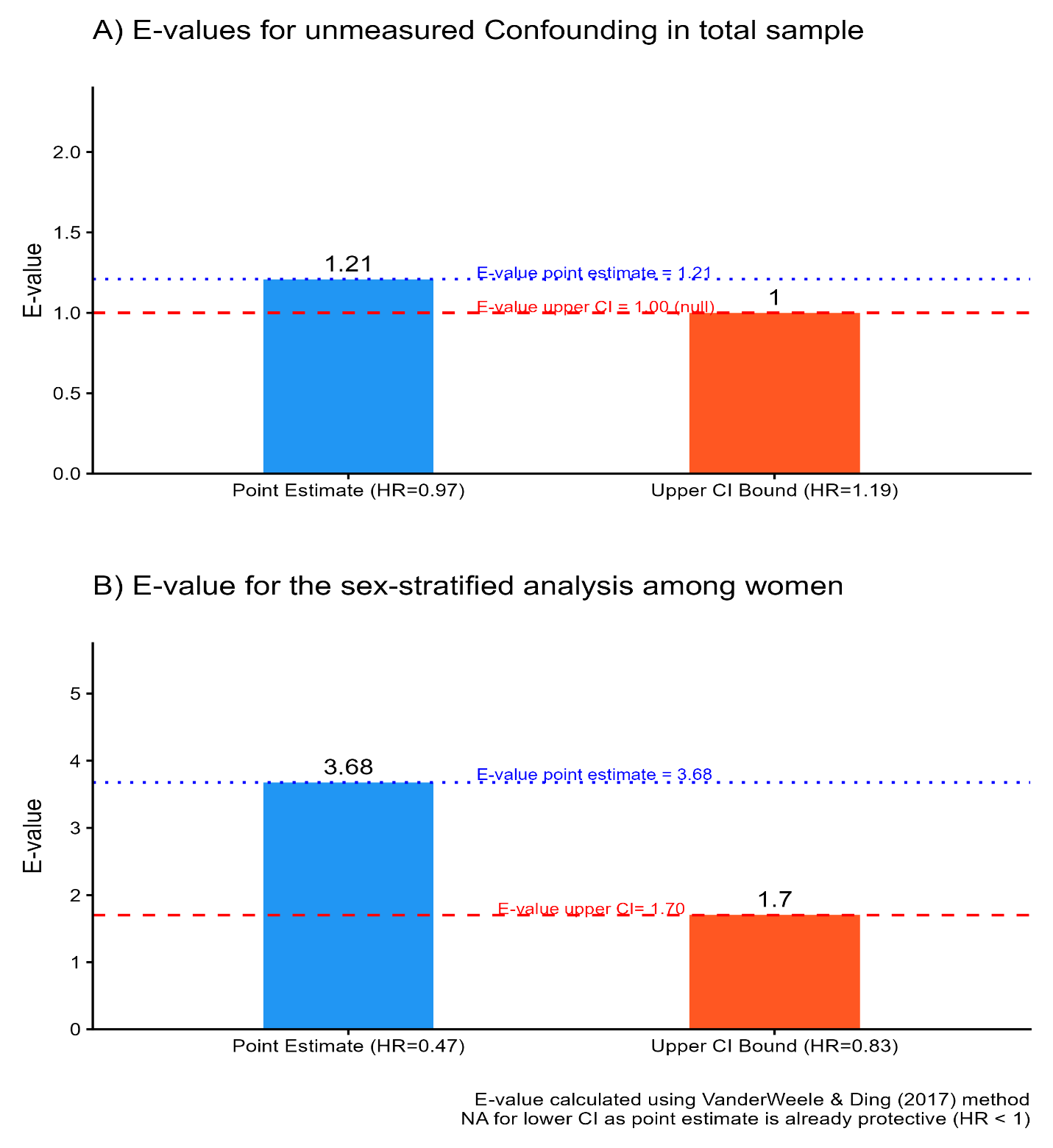


Figure S2. E-value for main results and sex-stratifed analysis.

| Table S1. Sensitive analysis of the interaction between MIND diet score and MET hours per week on dementia risk | | | | | |
| --- | --- | --- | --- | --- | --- |
| MIND Diet | Physical activity (MET hours/week) | | | | Within strata of MIND Diet |
|  | MET hrs./wk. ≥28 | | MET hrs./wk. < 28 | |  |
|  | N _cases_/N _total_ | HR (95%CI) | N _cases_/N _total_ | HR (95%CI) |  |
| MIND Diet ≥7.5 | 58/1474 | [Reference] | 95/1244 | 1.23 (0.88, 1.71) | 1.23 (0.88, 1.71) |
| MIND Diet <7.5 | 76/1039 | 1.62 (1.15, 2.29) | 136/1259 | 1.50 (1.09, 2.06) | 0.93 (0.69, 1.24) |
| Within strata of MET hrs./wk. | | 1.62 (1.15, 2.29) | 1.22 (0.93, 1.59) | |  |
| Measure of interaction on the additive scale:  RERI (95% CI): -0.35 (-1.14, 0.16);  AP (95% CI): -0.23 (-0.70, 0.13);  Synergy index (95% CI): 0.59 (0.28, 1.24).  Measure of interaction on multiplicative scale: HR (95% CI)= 0.75 (0.49, 1.16)  Model adjusted for: age, sex, education, employment status, smoking status, alcohol consumption, APOE4, depressive symptoms, antidiabetic medication, lipid-lowering therapy, and antihypertensive. | | | | | |

| Table S2. Baseline characteristics of the study population stratified by Sex | | | |
| --- | --- | --- | --- |
| Variables | Total, N (%) | Male | Female |
| Number of participants | 5016 (100%) | 2114 (42.1%) | 2902 (57.9%) |
| Number of cases | 365 (7.3%) | 139 (6.6%) | 226 (7.8%) |
| Age, Mean± SD | 69.76±8.73 | 71.0±8.9 | 68.7±8.5 |
| **MIND Diet** |  |  |  |
| MIND Diet <7.5 | 2298 (45.8%) | 1088 (51.5%) | 1210 (41.7%) |
| MIND Diet ≥7.5 | 2718 (54.2%) | 1026 (48.5%) | 1692 (58.3%) |
| **Physical activity** |  |  |  |
| MET h/week < 28.0 | 2503 (49.9% | 1020 (48.2%) | 1419 (48.9%) |
| MET h/week ≥ 28.0 | 2513 (50.1%) | 1094 (51.8%) | 1483 (51.1%) |
| **Education** |  |  |  |
| Primary | 392 (7.8%) | 126 (6.0%) | 266 (9.2%) |
| Lower/intermediate | 2001 (39.9%) | 539 (25.5%) | 1462 (50.4%) |
| Intermediate vocational | 1518 (30.3%) | 800 (37.8%) | 718 (24.7%) |
| Higher | 1105 (22.0%) | 649 (30.7%) | 456 (15.7%) |
| **Smoking status** |  |  |  |
| Non-smoker | 4465 (89.0%) | 1884 (89.1%) | 2581 (88.9%) |
| Current smokers | 549 (10.9%) | 229 (10.8%) | 320 (11.0%) |
| Total alcohol, Mean± SD | 7.40±8.09 | 10.1 (9.68) | 5.46 (6.01) |
| **Employment status** |  |  |  |
| Retired or unemployed | 4125 (82.2%) | 1604 (75.9%) | 2521 (86.9%) |
| Employed | 886 (17.7%) | 509 (24.1%) | 377 (13.0%) |
| **Sleep quality (PSQI)** |  |  |  |
| Good sleep (PSQI<5) | 3648 (72.7%) | 1803 (85.3%) | 1845 (63.6%) |
| Poor sleep (PSQI≥5) | 1368 (27.3%) | 311 (14.7%) | 1057 (36.4%) |
| **Depressive symptoms** |  |  |  |
| No depression (CESD<16) | 4603 (91.8%) | 2018 (95.5%) | 2585 (89.1%) |
| Depression (CESD≥16) | 413 (8.2%) | 96 (4.5%) | 317 (10.9%) |
| **APOE ε4 carrier** |  |  |  |
| APO ε4- | 3641 (72.6%) | 1541 (72.9%) | 2100 (72.4%) |
| APO ε4+ | 1375 (27.4%) | 573 (27.1%) | 802 (27.6%) |
| Body Mass Index (kg/m^2^) | 27.50±4.31 | 27.4 (3.60) | 27.5 (4.76) |
| Glucose levels, Mean± SD | 5.79±1.23 | 5.95 (1.32) | 5.67 (1.15) |
| Non-HDL cholesterol | 1.49±0.42 | 1.31 (0.350) | 1.63 (0.424) |
| **Antidiabetic medication** |  |  |  |
| No | 4569 (91.1%) | 1890 (89.4%) | 2679 (92.3%) |
| Yes | 447 (8.9%) | 224 (10.6%) | 223 (7.7%) |
| **Lipid-lowering therapy** |  |  |  |
| No | 3474 (69.3%) | 1344 (63.6%) | 2130 (73.4%) |
| Yes | 1542 (30.7%) | 770 (36.4%) | 772 (26.6%) |
| Systolic BP, Mean± SD | 144.63±22.20 | 146 (21.1) | 144 (23.0) |
| **Blood pressure-lowering medication** | | | |
| No | 4982 (99.3%) | 2098 (99.2%) | 2884 (99.4%) |
| Yes | 34 (0.7%) | 16 (0.8%) | 18 (0.6%) |
| Notes: SD=standard deviation, N=number of participants; PSQI, Pittsburgh Sleep Quality Index; CESD, Center for Epidemiologic Studies Depression Scale; HDL=high density lipoprotein; BP, blood pressure. | | | |

| Table S3. Sensitive analysis among participants with follow-up time of ≥ 3 years (N=4621) | | | | | |
| --- | --- | --- | --- | --- | --- |
| MIND Diet | Physical activity (MET hours/week) | | | | Within strata of MIND Diet |
|  | MET hrs./wk. ≥28 | | MET hrs./wk. < 28 | |  |
|  | N _cases_/N _total_ | HR (95%CI) | N _cases_/N _total_ | HR (95%CI) |  |
| MIND Diet ≥7.5 | 47/ 1393 | [Reference] | 77/ 1154 | 1.36 (0.94, 1.97) | 1.36 (0.94, 1.97) |
| MIND Diet <7.5 | 53/ 953 | 1.51 (1.02, 2.25) | 90/ 1121 | 1.38 (0.95, 2.0) | 0.91 (0.64, 1.3) |
| Within strata of MET hrs./wk. | | 1.51 (1.02, 2.25) | 1.02 (0.75, 1.39) | |  |
| Measure of interaction on the additive scale:  RERI (95% CI): -0.49 (-1.44, 0.1);  AP (95% CI): -0.35 (-0.97, 0.09);  Synergy index (95% CI): 0.44 (0.16, 1.18).  Measure of interaction on multiplicative scale: HR (95% CI)= 0.67 (0.41, 1.11).  Model adjusted for: age, sex, education, employment status, smoking status, alcohol consumption, APOE4, depressive symptoms, antidiabetic medication, lipid-lowering therapy, and antihypertensive. | | | | | |

| Table S4. Stratified interaction between MIND Diet and MET hours per week on dementia risk based on education level | | | | | | | | | | | |  |
| --- | --- | --- | --- | --- | --- | --- | --- | --- | --- | --- | --- | --- |
| Lower education (N=2393) | | | | | |  | Higher education (N=2623) | | | | | |
| MIND Diet | Physical activity (MET hours/week) | | | | Within strata of MIND Diet |  | Physical activity (MET hours/week) | | | | Within strata of MIND Diet | |
|  | MET hrs./wk. ≥28 | | MET hrs./wk. < 28 | |  |  | MET hrs./wk. ≥28 | | MET hrs./wk. < 28 | |  |  |
|  | N _cases_/N _total_ | HR (95%CI) | N _cases_/N _total_ | HR (95%CI) |  |  | N _cases_/N _total_ | HR (95%CI) | N _cases_/N _total_ | HR (95%CI) |  |  |
| MIND Diet ≥7.5 | 30/ 616 | 1.0 | 54/ 574 | 1.45 (0.92, 2.3) | 1.45 (0.92, 2.3) |  | 28/ 858 | 1.0 | 41/ 670 | 1.22 (0.74, 1.99) | 1.22 (0.74, 1.99) | |
| MIND Diet <7.5 | 41/ 505 | 1.64 (1.02, 2.65) | 75/ 698 | 1.35 (0.86, 2.11) | 0.82 (0.55, 1.23) |  | 35/ 534 | 1.66 (1.0, 2.76) | 61/ 561 | 1.95 (1.22, 3.12) | 1.17 (0.76, 1.81) | |
| Within strata of MET hrs./wk. | | 1.64 (1.02, 2.65) | 0.93 (0.65, 1.33) | | |  | 1.66 (1.0, 2.76) | |  | 1.61 (1.08, 2.4) | | |
| Measure of interaction on the additive scale:  RERI (95% CI): -0.74 (-2.09, -0.03);  AP (95% CI): -0.55 (-1.36, 0.02);  Synergy index (95% CI): 0.32 (0.1, 1.04).  Measure of interaction on multiplicative scale: HR (95% CI)= 0.57 (0.31, 1.03). | | | | | |  | Measure of interaction on the additive scale:  RERI (95% CI): 0.07 (-1.18, 0.92)  AP (95% CI): 0.04 (-0.55, 0.43);  Synergy index (95% CI): 1.08 (0.39, 3.04).  Measure of interaction on multiplicative scale: HR (95% CI)= 0.97 (0.51, 1.83) . | | | | | |
| Adjusted model 2: age, education, employment status, glucose levels, BMI, smoking status, alcohol consumption, systolic BP, non-HDL cholesterol, APOE4, depressive symptoms, poor sleep quality, antidiabetic medication, lipid lowering therapy, and antihypertensive. | | | | | | | | | | | |  |

| Table S5. Estimated effect of covariates on incident dementia | |
| --- | --- |
| Variables | HR (95%CI)^a^ |
| Age, Mean± SD | 1.14 (0.89, 1.16) |
| **Sex** | |
| Male | 1.0 |
| Female | 1.01 (0.78, 1.29) |
| **Education** | |
| Primary | 1.0 |
| Lower/intermediate | 0.95 (0.67, 1.35) |
| Intermediate vocational | 1.03 (0.71, 1.49) |
| Higher | 0.84 (0.54, 1.29) |
| **Smoking status** |  |
| Non-smoker | 1.0 |
| Current smokers | 1.37 (0.95, 1.99) |
| Total alcohol, Mean± SD | 0.99 (0.98, 1.01) |
| **Employment status** | |
| Retired or unemployed | 1.0 |
| Employed | 0.40 (0.27, 0.62) |
| **Sleep quality (PSQI)** | |
| Good sleep (PSQI<5) | 1.0 |
| Poor sleep (PSQI≥5) | 0.67 (0.53, 0.86) |
| **Depressive symptoms** | |
| No depression (CESD<16) | 1.0 |
| Depression (CESD≥16) | 1.01 (0.69, 1.46) |
| **APOE** **ε4 carrier** | |
| APO ε4- | 1.0 |
| APO ε4+ | 2.33 (1.88, 2.88) |
| Body Mass Index (kg/m^2^) | 0.96 (0.94, 0.99) |
| Glucose levels, Mean± SD | 1.16 (1.05, 1.27) |
| Non-HDL cholesterol | 1.12 (0.84, 1.49) |
| **Antidiabetic medication** | |
| No | 1.0 |
| Yes | 0.95 (0.62, 1.46) |
| **Lipid-lowering therapy** | |
| No | 1.0 |
| Yes | 0.98 (0.78, 1.23) |
| Systolic BP, Mean± SD | 0.99 (0.99, 1.00) |
| **Blood pressure-lowering medication** | |
| No | 1.0 |
| Yes | 0.48 (0.07, 3.43) |
| Notes: SD=standard deviation, N=number of participants; PSQI, Pittsburgh Sleep Quality Index; CESD, Center for Epidemiologic Studies Depression Scale; HDL=high density lipoprotein; BP, BLOOD PRESSURE.  ^a^Adjusted model 2: age, education, employment status, glucose levels, BMI, smoking status, alcohol consumption, systolic BP, non-HDL cholesterol, APOE4, depressive symptoms, poor sleep quality, antidiabetic medication, lipid-lowering therapy, and antihypertensive. | |

**Table S6.** STROBE Statement—checklist of items that should be included in reports of observational studies

|  | **Item No** | **Recommendation** | **Page  No** |
| --- | --- | --- | --- |
| **Title and abstract** | 1 | (*a*) Indicate the study’s design with a commonly used term in the title or the abstract | 1-2 |
|  |  | (*b*) Provide in the abstract an informative and balanced summary of what was done and what was found | 2 |
| **Introduction** | | | |
| Background/rationale | 2 | Explain the scientific background and rationale for the investigation being reported | 3 |
| Objectives | 3 | State specific objectives, including any prespecified hypotheses | 4 |
| **Methods** | | | |
| Study design | 4 | Present key elements of study design early in the paper | 5 |
| Setting | 5 | Describe the setting, locations, and relevant dates, including periods of recruitment, exposure, follow-up, and data collection | 5 |
| Participants | 6 | (*a*) *Cohort study*—Give the eligibility criteria, and the sources and methods of selection of participants. Describe methods of follow-up  *Case-control study*—Give the eligibility criteria, and the sources and methods of case ascertainment and control selection. Give the rationale for the choice of cases and controls  *Cross-sectional study*—Give the eligibility criteria, and the sources and methods of selection of participants | 5 |
|  |  | (*b*) *Cohort study*—For matched studies, give matching criteria and number of exposed and unexposed  *Case-control study*—For matched studies, give matching criteria and the number of controls per case | - |
| Variables | 7 | Clearly define all outcomes, exposures, predictors, potential confounders, and effect modifiers. Give diagnostic criteria, if applicable | 5 |
| Data sources/ measurement | 8* | For each variable of interest, give sources of data and details of methods of assessment (measurement). Describe comparability of assessment methods if there is more than one group | *5* |
| Bias | 9 | Describe any efforts to address potential sources of bias | 8 |
| Study size | 10 | Explain how the study size was arrived at | 5 |
| Quantitative variables | 11 | Explain how quantitative variables were handled in the analyses. If applicable, describe which groupings were chosen and why | 8 |
| Statistical methods | 12 | (*a*) Describe all statistical methods, including those used to control for confounding | 8 |
|  |  | (*b*) Describe any methods used to examine subgroups and interactions | 8 |
|  |  | (*c*) Explain how missing data were addressed | 8 |
|  |  | (*d*) *Cohort study*—If applicable, explain how loss to follow-up was addressed  *Case-control study*—If applicable, explain how matching of cases and controls was addressed  *Cross-sectional study*—If applicable, describe analytical methods taking account of sampling strategy | 8 |
|  |  | (*e*) Describe any sensitivity analyses | 8 |

Continued on next page

| **Results** | | | |
| --- | --- | --- | --- |
| Participants | 13* | (a) Report numbers of individuals at each stage of study—eg numbers potentially eligible, examined for eligibility, confirmed eligible, included in the study, completing follow-up, and analysed | 9 |
|  |  | (b) Give reasons for non-participation at each stage | - |
|  |  | (c) Consider use of a flow diagram | 5 |
| Descriptive data | 14* | (a) Give characteristics of study participants (eg demographic, clinical, social) and information on exposures and potential confounders | 9 |
|  |  | (b) Indicate number of participants with missing data for each variable of interest | 7 |
|  |  | (c) *Cohort study*—Summarise follow-up time (eg, average and total amount) | 2 |
| Outcome data | 15* | *Cohort study*—Report numbers of outcome events or summary measures over time | *9-10* |
|  |  | *Case-control study—*Report numbers in each exposure category, or summary measures of exposure | *-* |
|  |  | *Cross-sectional study—*Report numbers of outcome events or summary measures | *-* |
| Main results | 16 | (*a*) Give unadjusted estimates and, if applicable, confounder-adjusted estimates and their precision (eg, 95% confidence interval). Make clear which confounders were adjusted for and why they were included | 9-10 |
|  |  | (*b*) Report category boundaries when continuous variables were categorized | 10 |
|  |  | (*c*) If relevant, consider translating estimates of relative risk into absolute risk for a meaningful time period | - |
| Other analyses | 17 | Report other analyses done—eg analyses of subgroups and interactions, and sensitivity analyses | 10 |
| **Discussion** | | | |
| Key results | 18 | Summarise key results with reference to study objectives | 10 |
| Limitations | 19 | Discuss limitations of the study, taking into account sources of potential bias or imprecision. Discuss both direction and magnitude of any potential bias | 12 |
| Interpretation | 20 | Give a cautious overall interpretation of results considering objectives, limitations, multiplicity of analyses, results from similar studies, and other relevant evidence | 12 |
| Generalisability | 21 | Discuss the generalisability (external validity) of the study results | 12 |
| **Other information** | | | |
| Funding | 22 | Give the source of funding and the role of the funders for the present study and, if applicable, for the original study on which the present article is based | 14 |

*Give information separately for cases and controls in case-control studies and, if applicable, for exposed and unexposed groups in cohort and cross-sectional studies.

**Note:** An Explanation and Elaboration article discusses each checklist item and gives methodological background and published examples of transparent reporting. The STROBE checklist is best used in conjunction with this article (freely available on the Web sites of PLoS Medicine at http://www.plosmedicine.org/, Annals of Internal Medicine at http://www.annals.org/, and Epidemiology at http://www.epidem.com/). Information on the STROBE Initiative is available at www.strobe-statement.org.
